# Quality of biosafety guidelines for dental clinical practice throughout the world in the early COVID-19 pandemic: a systematic review

**DOI:** 10.4178/epih.e2021089

**Published:** 2021-10-22

**Authors:** Renata Cristina Soares, Juliana Schaia Rocha, Saulo Vinicius da Rosa, Jéssica Rodrigues da Silva Noll Gonçalves, Priscilla Lesly Perlas Condori, Ana Elisa Ribeiro, Samuel Jorge Moysés, Márcia Helena Baldani

**Affiliations:** 1 Department of Dentistry, State University of Ponta Grossa, Paraná, Brazil; 2 Department of Dentistry, Pontifical Catholic University of Paraná, Paraná, Brazil; 3 Public Service – Pinhais, Paraná, Brazil

**Keywords:** Dentistry, COVID-19, SARS-CoV-2, Biosafety, Guidelines

## Abstract

**OBJECTIVES:**

To conduct a systematic review of coronavirus disease 2019 (COVID-19)-related biosafety guidelines for dental clinical practice in the early stage of the pandemic, focusing on quality assessment.

**METHODS:**

Electronic (via PubMed, Scopus, Web of Science, Latin American and Caribbean Health Sciences Literature database, Brazilian Library in Dentistry, and Cochrane Library) and gray literature searches were performed for documents published up to May 12, 2020. Guidelines updated until April 17, 2021 were identified. Documents were included as guidelines if they (1) consisted of a set of statements, directions, or principles presenting current or future rules or policy; (2) were developed by government agencies, institutions, organizations, or expert panels; and (3) were related to the general conduct of healthcare activities rather a particular condition. Two researchers, using the Appraisal of Guidelines for Research & Evaluation II, independently extracted the recommendations and evaluated the quality of the guidelines.

**RESULTS:**

Twenty-seven documents from 19 countries were included in the review. These documents presented 122 recommendations related to (1) professional biosafety; (2) patients’/companions’ safety; (3) the organization and biosafety of the physical dental facility environment; and (4) the work process in dental care. Overall, the scientific quality of the guidelines was considered low. Some recommendations presented in these guidelines would require further research to establish their effectiveness.

**CONCLUSIONS:**

We found a wide variety of biosafety guidelines for dental practice regarding COVID-19 in the early months of the pandemic, but their quality was low. Biosafety recommendations should be frequently updated.

## INTRODUCTION

Since the first cases of coronavirus disease 2019 (COVID-19) were reported as viral pneumonia with an unknown cause in December 2019, COVID-19 has become a pandemic and spread across the world [1]. On January 8, 2020, the World Health Organization declared the outbreak to be a public health emergency of international interest [2,3], and it was officially declared a pandemic on March 11, 2020 [4]. COVID-19 is a disease caused by severe acute respiratory syndrome coronavirus 2 (SARS-CoV-2). People with mild COVID-19 might experience cough, sore throat, fever, diarrhea, headache, muscle or joint pain, fatigue, and a loss of sense of smell and taste. Symptoms of COVID-19 pneumonia include breathlessness, loss of appetite, confusion, pain or pressure in the chest, and a high temperature (above 38°C) [5]. The signs are evaluated by clinical examinations, including lung sounds, blood pressure, and heart rate [5,6].

Those who have prolonged close contact with people with COVID-19, whether symptomatic or asymptomatic, are at a higher risk of infection [3,6-9]. There is evidence that the virus can be transmitted through direct and/or indirect contact, including saliva [10-13]. The virus can also be spread through the air, through saliva droplets and aerosols originating from clinical procedures [14].

Dental procedures can generate large numbers of droplets and aerosols [3], and studies have shown that the virus can stay alive in aerosols for up to 3 hours after treatment; on surfaces, the virus can remain alive for days [15]. Therefore, due to the characteristics of the dental office environment, the risk of contagion due to the exposure to saliva, blood, and aerosol/droplet production during most dental procedures is high [16,17]. In addition, some procedures and treatments take a long time to complete, contributing to a high risk of contamination [3,18]. Given these circumstances, the usual protective measures might not be sufficient to prevent the spread of the disease in dental offices.

In this context, dentists and staff should adopt individual protective measures during dental care delivery, as well as avoiding or minimizing procedures that generate aerosols to prevent and control the infection [3]. Since the beginning of the COVID-19 pandemic, stakeholders at dental institutions and government agencies urgently formulated and made available strict biosafety and infection control guidelines for dental care, providing recommendations on biosafety within the context of dental services [19-21].

However, due to the large amount of information published on the topic within a few months, and the rapid emergence of new evidence, it is difficult to identify which guidelines and recommendations are based on best practices to prevent the spread of COVID-19 in the dental environment. In fact, a brief review including national guidelines for health services produced in the very beginning of the pandemic (until March 2020) showed poor overall quality for most of them, with a lack of clear links between the recommendations and scientific evidence [22].

A review of the guidance issued by international organizations and professional bodies regarding the re-opening of dental services showed considerable variation in the safety procedures required. All sources emphasized the need to focus on activities that minimize risk to staff, patients and the public, and to support high-quality clinical care [23]. Since systematic literature reviews are the studies of choice for collecting and summarizing the extant evidence on a given issue, the aim of this research was to conduct a review of COVID-19 related biosafety guidelines for dental clinical practice in the early stage of the pandemic, focusing on quality assessment.

## MATERIALS AND METHODS

### Search strategy

This research followed a protocol based on the PRISMA-P (Preferred Reporting Items for Systematic Reviews and Meta-Analysis Protocols) guidelines [24] and was registered on the PROSPERO platform under the registration number CRD42020185641.

The study question was: “What were the biosafety guidelines used in dental clinics for the prevention and control of COVID-19 during the early stage of the pandemic?”

The search strategy was carried out in the following electronic databases: PubMed, Scopus, Web of Science, Latin American and Caribbean Health Sciences Literature database, Brazilian Library in Dentistry, and Cochrane Library. To complement the search for relevant documents, the gray literature was searched through Google Scholar, Google, and websites of societies and health institutions worldwide (Supplementary Material 1). Subsequently, manual searches were performed in the references of the included publications to identify any additional important documents that were not recovered in the electronic search.

The search strategy included a controlled vocabulary (Medical Subject Headings [MeSH] terms and Health Sciences Descriptors [DeCS]) and other free keywords (uncontrolled vocabulary), as well as the logical search operators “AND” and “OR,” and was adapted for each database. The combination of terms was defined based on the study question and the population–intervention– comparison–outcome–study design (PICO-S) framework (Supplementary Material 2):

Population (P): dentistry, dentists, oral health teams, dental offices; Intervention (I): outpatient biosafety measures/strategies; Comparison (C): not applicable; Outcome (O): prevention and control of COVID-19; Study design (S): recommendations, guides, guidelines, technical guidelines, orientations, protocols, manuals, technical notes, strategic plans, notifications, and contingency plans.

### Eligibility criteria

The search in the databases did not apply language restrictions and included documents published in the early COVID-19 pandemic, corresponding to the period after the appearance of the first confirmed case until May 12, 2020. We considered eligible for inclusion all publications that were characterized as recommendations, guides, guidelines, technical guidelines, orientations, protocols, manuals, technical notes, strategic plans, notifications, and contingency plans that (1) were issued by a competent body or agency; (2) had references; and (3) involved other health professionals and enabled the extraction of specific data from dentistry. To be included, the publication was required to meet the criteria that define a “guideline” according to the MeSH, which stipulate that a guideline (1) consists of a set of statements, guidelines or principles that present the present or future rules or policies; (2) is developed by government agencies, institutions, organizations, or expert panels; and (3) is related to the general conduct of health activities, and not to a particular condition.

The exclusion criteria were: (1) literature reviews; (2) letters to the editor; (3) opinions; (4) primary studies that evaluated biosafety measures in dentistry; (5) other research articles; (6) documents that did not fall under the category of guidelines and could not be evaluated using the evaluation instrument; and (7) documents that were not available after the usual forms of search and retrieval had been exhausted.

Publications that met the inclusion criteria were summarized for further evaluation and data extraction. All relevant documents included for analysis were translated and revised. We also conducted a search on Google and/or the websites of institutions that published the included guidelines to identify the dates of updates to the documents until April 17, 2021.

### Study selection and data collection

We used the EndNote reference manager. Initially, 2 researchers independently scrutinized the documents by title and abstract, according to the research strategy described above. Inter-rater reliability was calculated at this stage using the Cohen kappa statistic, obtaining a value of 0.71, which was considered to indicate substantial reliability. To determine whether studies would be included or excluded, the full texts were then evaluated by 2 reviewers independently. The results were compared to check for divergences, which were resolved through discussion and consensus. A third reviewer was consulted to resolve any additional differences, checking for any bias from the evaluators.

Data collection was performed by 5 calibrated researchers who used a structured data extraction form that was submitted to a pilot process. Each manuscript was reviewed by 2 researchers on the team, who independently extracted data on the biosafety recommendations and performed the guideline quality assessment. Any disagreement or possible interpretative differences were solved by consulting the entire team to obtain consensus.

### Synthesis of the biosafety recommendations

We performed a descriptive analysis, summarizing the main characteristics of the guidelines and organizing a synthesis of the main recommendations, which were listed according to 4 predefined categories or themes, as follows: (1) professional biosafety; (2) patients’/companions’ safety; (3) the organization and biosafety of the physical dental facility environment; and (4) the work process in dental care.

### Assessment, analysis, and synthesis of the quality of the guidelines

We used the Brazilian Portuguese version of the Appraisal of Guidelines for Research and Evaluation II (AGREE II) instrument [25,26]. Inter-rater reliability was calculated using the Fleiss kappa statistic [27] obtaining a value of 0.58, which indicates intermediate to good reliability. The AGREE II consists of 23 key items organized within 6 domains followed by 2 global rating items (“Overall Assessment”) [28,29].

### Ethics statement

As this article is a systematic review, approval by an ethics committee was not required.

## RESULTS

**Figure 1.**
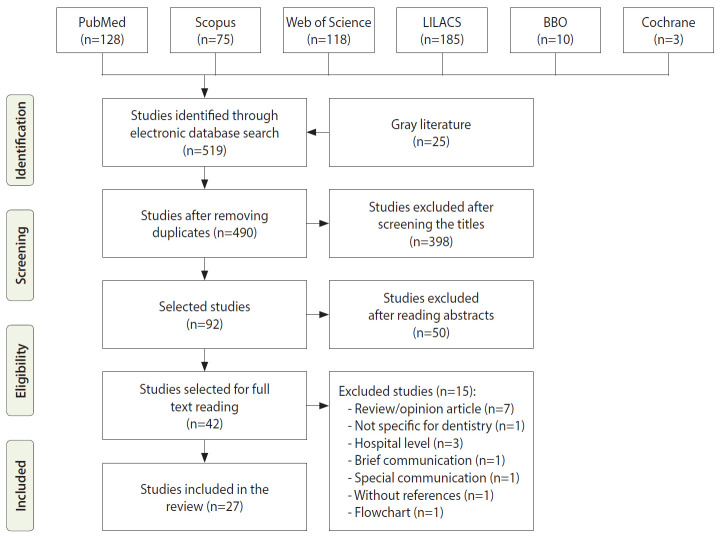
Flowchart of the systematic review search process. LILACS, Latin American and Caribbean Health Sciences Literature database; BBO, Brazilian Library in Dentistry.

We identified 519 records through the database search and a further 25 through gray literature searches (Figure 1). After the exclusion of duplicates, 490 records were screened based on their title and abstract, and 42 of those had their full text retrieved for analysis. A careful reading of the full texts led to the exclusion of 15 documents, while 27 [30-56] of them remained and met the eligibility criteria, for which reason they were included in the review. The reasons for excluding texts that were evaluated in full are presented in Supplementary Material 3.

We found guidelines originating from all continents, and most guidelines were published in upper-middle or high-income countries. We did not find guidelines produced in low-income countries. Government agencies and dental organizations from countries in Europe and Latin America produced most of the publications that were mainly referred to as protocols or recommendations. In the search for updates, we found 15 guidelines that had been updated by the date of April 17, 2021 (Table 1).

Thorough scrutiny of the 27 documents included led to a summary of 122 recommendations regarding biosafety for dental practice in the face of COVID-19 (Table 2): 26 recommendations were related to professional biosafety, 32 dealt with the safety of the patients and their companions, 46 provided directions on the organization of the dental office environment regarding biosafety measures, and 18 presented information on how to conduct dental care (techniques, interventions, procedures, and materials).

**Figure 2.**
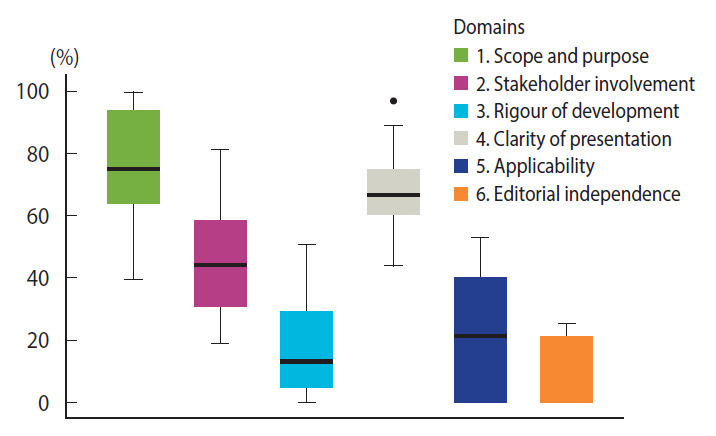
Combined Appraisal of Guidelines for Research and Evaluation II assessment for all the coronavirus disease 2019 biosafety guidelines for dental practice (n=27). Vertical lines indicate range (% adequacy^1^); horizontal line represents mean adequacy score^1^ for each domain. ^1^Percentage of maximum possible score per domain.

Figure 2 summarizes the distribution of the adequacy of the guidelines—that is, the percentage of the maximum possible score for each item in the 6 AGREE II domains. Higher mean adequacy scores were observed for a clear indication of the scope and purpose of the guidelines, as well as for clarity of presentation. However, fewer items in the rigor of development domain showed high scores, and many of the guidelines had no indication of editorial independence. Table 3 demonstrates the individual and overall scores of each guideline for the domains. Details on the specific scores for each item are shown in Supplementary Material 4.

### Domain 1: scope and purpose

The scores for domain 1 ranged from 38.9% to 100%. In general, the guidelines showed good to excellent scores, with a median value of 75.0% and a mode of 72.0%. Two documents received adequacy scores lower than 50.0% for domain 1: those published by the Ministry of Health of Argentina (38.9%) [34] and American Dental Association, United States (44.4%) [47]. Three documents had an adequacy score of 100%, from South Africa [30], Germany [31], and Spain [46].

### Domain 2: stakeholder involvement

The scores for this domain ranged from 19.4% to 80.5%. In general, the guidelines exhibited low scores, with a median value of 44.0% and a mode of 30.0%. All guidelines scored 1 (the lowest possible) for the item related to considering the viewpoints and preferences of the target population (e.g., patients or the public) when defining the recommendations. The highest-scoring guideline for domain 2 was published by the College of Dental Surgeons of Costa Rica (80.5%) [41].

### Domain 3: rigor of development

This domain showed the poorest results; the scores ranged from 0.0% to 50.0%, with a median of 12.5% and a mode of 4.2%. The most adequate guidelines in this domain originated from Spain (51.0%) [46], South Africa (50.0%) [30] and Germany (41.7%) [31]. The documents from the Ministry of Health of Paraguay [54], Costa Rica [42] and Chile [44] had the lowest scores.

Regarding the individual items in domain 3, most of the guidelines did not refer to systematic methods to search for evidence (item 7) and did not clearly describe the strengths and limitations of the body of evidence (item 9). The highest score for these items was 4. Regarding a clear description of the criteria used to select the evidence (item 8), only the guideline developed by the Society of Dentists and Stomatologists of Catalonia (Spain) [46] scored high; for the other guidelines, the scores ranged between 1 and 2. The guidelines developed in Belgium [35] and Spain [46] had the maximum score for item 11, whereas most of the other guidelines had a score of 1. For item 13, which related to whether the guideline was externally reviewed by experts prior to its publication, only 3 guidelines obtained the highest points possible (a score of 7): the document from South Africa [30], a publication of the Ministry of Health of Paraguay [53] and the guideline from the Ministry of Health and Social Protection of Colombia [40]. The document from Catalonia (Spain) [46] scored 4, and all the others scored 1 and 2.

### Domain 4: clarity of presentation

Domain 4 received scores ranging from 44.4% to 97.2%, with a median of 12.5% and a mode of 4.2%. In general, the guidelines had good to excellent scores, with a median value and mode of 66.7%. The guideline from New Zealand [52] had the lowest score (44.4%), and the publication from Catalonia (Spain) [46] had the highest (97.2%). Other documents that received scores higher than 80.0% for domain 4 were published by the American Dental Association (United States) [47], Indian Dental Association (India) [50], Collegiate Organization of Dentists of Spain (Spain) [45], German Society of Dentists (Germany) [31] and Federal Council of Dentistry (Brazil) [36].

### Domain 5: applicability

The scores for domain 5 ranged from 0.0% to 50.0%, with median of 20.8%. Most of the documents surveyed presented low scores in this domain. Seven documents had a score of 0.0%, and only 2 guidelines had an adequacy score of 50.0% or higher; these studies were from Portugal [55] and Belgium [35].

### Domain 6: editorial independence

The guidelines were less than suitable in this domain, with scores ranging from 0.0% to 25.0%. Sixteen documents scored 0.0% for adequacy in this domain, and none of the 27 guidelines had complete information on how this issue was addressed.

### Overall assessment

The overall evaluation of the guidelines ranged from 3 to 7. The highest scores were attributed to the guidelines developed by the Society of Dentists and Stomatologists of Catalonia (Spain) [46], the German Society of Dentists (Germany) [31] and the Federal Council of Dentistry (Brazil) [36]. The lowest score was found for the guideline developed by the Ministry of Health of Chile [44].

## DISCUSSION

Although dentistry is considered a high-exposure occupation, the risk of infection by dental professionals, as well as other healthcare workers, has been a major concern since the start of the COVID-19 pandemic [6]. According to recent reviews, there is not enough evidence to draw firm conclusions regarding the relationship between healthcare occupational exposure and the COVID-19 pandemic [57,58]. However, systematic reviews have suggested that health professionals are not at an increased risk of infection compared to the overall population if appropriate personal protective equipment is used and there is appropriate adherence to enhanced infection control and prevention precautions [57,58].

This systematic review identified a wide variety of biosafety recommendations for the prevention and control of COVID-19 in dentistry. However, the findings showed that the guidelines had limitations, mainly in relation to the rigor of development, lack of clarification on financing, conflicts of interest, and advice and/or tools on how the recommendations can be put into practice. Moreover, we were unable to identify any revisions or updates to 12 of the 27 guidelines.

With growing global concerns about controlling the spread of SARS-CoV-2 in the dental clinic, a comprehensive understanding of biosafety measures based on robust scientific evidence is essential for effective prevention/control of COVID-19 in this context.

The limited level of rigor in the construction of the guidelines made it difficult to appreciate the remarkable variation in the recommendations. For instance, the wide variation in recommendations on materials suitable for cleaning and disinfecting surfaces (alcohol from 60.0 to 80.0%, soap and detergent, 0.1% benzalkonium chloride, 0.5% hydrogen peroxide, 0.1% or more sodium hypochlorite, disinfectants based on quaternary ammonium, disinfectants based on chlorine, 2.0% glutaraldehyde).

In relation to patient care regarding pre-procedure mouthwashes, the recommendation most often cited in the guidelines was to use hydrogen peroxide at concentrations between 0.5% and 1.5% [30-39,41-44,47-54]. Although povidone-iodine (PVP-I) was less frequently recommended [30,32,34,35,37-39,41,43,47-53] than hydrogen peroxide in the included guidelines, the evidence indicates that PVP-I is effective against SARS-CoV-2 [59,60].

The main care recommendations for professionals that stood out in relation to the usual biosafety measures were the use of N95, PFF2, PFF3, or equivalent masks and the use of facial shields [30-41,43-45,48,49,51,54-56]. The recommendations that gained the most prominence in general and in the waiting room and other dental clinic settings were keeping a safe distance [30,32-36,39-47,49-52,56] and removing objects to prevent the spread of the virus [30-32,34,35,41-47,49-52]. The most frequently indicated biosafety recommendations regarding dental care were related to the use of power suction/aspiration to reduce the amount of saliva in the oral cavity [30-37,40,41,44,46-56], the use of absolute isolation with a rubber dam [30,31,34,36,37,41-44,46-52,55,56], and performing procedures with 4 hands [30,34,36,37,40,43,44,46-51,53].

Most guidelines were produced in high-income or upper-middle-income countries. This result confirms the findings by Dagens et al. [22], so we can assume that some recommendations or technologies may not be realistic in low-income settings. For instance, performing screening with sophisticated and expensive technological tools, using an air purifier, and performing procedures with 4 hands. This inequality of contexts must be addressed, so that biosafety measures can be adapted to settings and environments where fewer resources are available.

There was substantial variability among quality domains within and across guidelines. This systematic review reveals that among the 6 domains evaluated by the AGREE II tool, 2 of them (scope and purpose, clarity of presentation) received the highest scores. This finding is consistent with systematic reviews that evaluated guidelines [61-63] suggesting that this component of guideline development may be easier to achieve or more highly valued by guideline development organizations [64].

Rigor of development, editorial independence, and applicability remain challenges. In general, the guidelines did not present the methods used for the development of the guidelines or presented this information superficially. It is important to highlight that the highest score related to domain 3 (rigor of development) was 51%, attributed to the guideline developed in Spain [46]. Item 14 of the AGREE II instrument, for the rigor of development domain, points out the importance of guidelines presenting current evidence. Thus, it is essential to have a procedure to update these documents. In this research, most of the guidelines presented low scores for this item. Another important point to be highlighted is that we faced a series of difficulties in identifying guideline updates, such as websites organized in a way that makes the search difficult and the availability of guidelines on websites that did not clearly indicate whether a guideline was an update of a previously published document or a new document. We observed that some guidelines did not indicate how many updates the document had; instead, they only identified the last update date. Therefore, some of these aspects can be considered for the planning and dissemination of guideline updates to facilitate the interpretation and identification of these documents by the target audience.

The findings of this review also suggest that guideline developers did not pay sufficient attention to the applicability of their recommendations, their target audiences, and implementation issues. The highest score for this domain was 52.8%, credited to the guideline developed in Portugal [55].

There are strengths and limitations of AGREE II that should be cited. This instrument offers the opportunity to conduct a systematic, specific, and objective evaluation of the quality of guidelines from all specialties, due to the wide range of domain items [65]. However, several researchers have indicated that these assessments are subjective [59,66,67]. The results of an AGREE II appraisal should be viewed with caution, as different guideline reviewers may interpret the items and scoring system differently [68]. Because of the aspects mentioned above, some researchers have reported that the AGREE II allows unclear distinctions between high-quality and low-quality guidelines [63,69,70]. However, despite its limitations, the AGREE II tool is relevant to a wide variety of health professionals, geographical areas, and guideline development processes [29,71].

As there is no threshold to establish the quality level of a guideline, these decisions should be shaped by the context in which the guideline is to be used and by evaluating the importance of the different domains and items in that context [25]. The guidelines presented here were developed under conditions of uncertainty at a time of international crisis. Due to the risk of contamination in dental environments, the COVID-19 pandemic required fast adaptation by dentists and decision-makers around the world. This situation was reflected in the rapid formulation of guidelines for the prevention/control of the spread of SARS-CoV-2 in dental clinics, which may have influenced the quality and, consequently, influenced the scores for AGREE II items. Considering these limitations, future guidelines should be devised with more rigorous methods, ensuring clarifications on financing, conflicts of interest, advice and/or tools on how the recommendations can be put into practice.

There are some limitations associated with this review. First, the dynamic nature and rapidity of information development in the context of a pandemic can lead to sudden changes in the recommendations for the prevention of COVID-19 infection in the dental environment. We acknowledge and appreciate that more guidelines have emerged since the early pandemic and that some guidelines included in this review have been updated. Secondly, the guidelines were published in a range of languages. We used fluent translators, but we also had to make use of translation software for some languages. This fact risks losing the finer nuances of a complex topic, although we believe that some idiomatic inaccuracy does not compromise the main aspects that counted in this review, as this is a scientific challenge found in all times and cultures. Third, systematic reviews on biosafety measures and protocols in dentistry have already been published [72-74], a fact that may reflect some overlapping with the information in the material presented in this review. However, those reviews did not assess the quality of the selected guidelines; this is the first systematic review evaluating the quality of biosafety guidelines in dental clinics for the prevention/control of COVID-19. Finally, the AGREE II tool was developed to evaluate guidelines produced by major teams in non-urgent conditions. Despite these limitations, this systematic review sought to follow judicious methods for the identification of guidelines developed worldwide by the established deadline, as well as to carefully evaluate the quality of their development. We have pointed out existing recommendations and possible areas to be improved in relation to the biosafety recommendations for the control and prevention of COVID-19 in dental clinics.

As the COVID-19 pandemic grows, biosafety guidelines in dentistry will be in increasing demand globally. Expecting strongly evidence-based interventions for a recently emerged disease is unreasonable, and it is likely that more thorough and numerous guidelines will be reviewed or produced as the pandemic progresses [22]. However, we need guidelines that are evidence-based, while also conveying to which populations and indications the guidance applies. When no evidence is available, this should also be made clear [22]. Due to the variations in the recommendations identified in this systematic review, and in other research that evaluated guidelines during this pandemic [19,22], the importance of a gold standard framework for guideline construction under conditions of uncertainty is evident [22].

The COVID-19 pandemic brought challenges to infection control in dental clinics. The post-pandemic context will also be challenging; therefore, the standardized biosafety measures already in use should be constantly reevaluated to identify the need to incorporate the professional measures that emerged during this pandemic into practice.

## CONCLUSION

The biosafety guidelines for the prevention and control of COVID-19 that emerged in the early months of the pandemic contained a wide variety of recommendations, but the quality of some guidelines was insufficient. The studied guidelines were not very homogeneous, preventing the formation of a robust scientific consensus and hindering the ability to offer safer and more reliable procedures. In addition, some recommendations require more studies on their effectiveness. Therefore, further guidelines for dental practice are needed with more clarification of the strictness of development, financing, conflicts of interest, and applicability. In view of the uncertainties generated by COVID-19, with many complex factors and mechanisms still unknown, biosafety recommendations should be updated as new high-quality evidence emerges.

## Figures and Tables

**Table 1.** Availability of coronavirus disease 2019 biosafety guidelines for dental practice, by date, origin, type of document and resources of the setting according to the World Bank classification (2020)

Organization	Document type	Country	Publication included in the systematic review	Versions/publications updated by Apr 17, 2021	Income group			
					Low	Lower middle	Upper middle	High
The South African Dental Association	Protocol	South Africa	May 10, 2020	Not found	-	-	○	-
Institute of German Dentists	Protocol	Germany	Apr 24, 2020	Not found	-	-	-	○
Latin American Association of Pediatric Dentistry	Recommendation	Latin America	2020	Jan 17, 2021	-	-	○	-
Austrian Dental Association	Guideline	Austria	Apr 30, 2020	Sep 30, 2020	-	-	-	○
Ministry of Health	Recommendation	Argentina	Apr 2, 2020	Jun 10, 2020	-	-	○	-
Dentistry Council	Protocol	Belgium	May 24, 2020	Apr 19, 2020	-	-	-	○
Federal Council of Dentistry	Recommendation	Brazil	Mar 25, 2020	01-Jun-20	-	-	○	-
				22-Jun-20				
				05-Apr-21				
Ministry of Health/ National Health Surveillance Agency	Technical note	Brazil	May 8, 2020	27-Oct-20	-	-	-	-
				25-Feb-21				
Straumann Group	Manual	Brazil	Apr 2020	Not found	-	-	-	-
Colombian Association of Faculties of Dentistry, Colombian Federation of Dentistry and Colombian Society of Dental Surgeons	Protocol	Colombia	May 7, 2020	Apr 23, 2020	-	-	○	-
Ministry of Health and Social Protection	Guidance	Colombia	04-May-20	May 14, 2020	-	-	-	-
Ministry of Public Health	Technical guideline	Costa Rica	27-Mar-20	12-Aug-20	-	-	○	-
College of Dental Surgeons	Recommendation	Costa Rica	14-Apr-20	Not found	-	-	-	-
Ministry of Health	Guidance	Chile	Jul 21, 2020	Not found	-	-	-	○
School of Dentistry/University of Chile	Recommendation	Chile	Apr 16, 2020	Oct, 2020	-	-	-	-
Collegiate Organization of Dentists of Spain	Strategic plan	Spain	May 1, 2020	Jun 12, 2020	-	-	-	○
Official College of Dentists and Stomatologists of Catalonia	Recommendation	Spain	Apr 30, 2020	May 6, 2020	-	-	-	-
				12-Feb-21				
American Dental Association	Protocol	USA	Apr 1, 2020	Mar 30, 2021	-	-	-	○
Ministry of Public Health	Protocol	Ecuador	2020	Not found	-	-	○	-
Stomatological School of Guatemala	Protocol	Guatemala	May 2020	Not found	-	-	○	-
Indian Dental Association	Protocol	India	Apr 20, 2020	Not found	-	○	-	-
School of Dentistry/ National Autonomous University of Mexico	Guidance	Mexico	Jun 15, 2020	Not found	-	-	○	-
Ministry of Health	Guideline	New Zealand	May 11, 2020	Mar 10, 2021	-	-	-	○
Ministry of Public Health and Social Welfare	Protocol	Paraguay	Apr 14, 2020	Not found	-	-	○	-
Ministry of Public Health and Social Welfare	Protocol	Paraguay	May 7, 2020	Not found	-	-	-	-
The Directorate-General of Health	Guidance	Portugal	May 1, 2020	July 20, 2020	-	-	-	○
Dental Association of Thailand	Protocol	Thailand	Apr 21, 2020	Not found	-	-	○	-

**Table 2.** Summary of the recommendations on COVID-19 biosafety for dental practice and percentage of the reviewed guidelines in which they appear (n=27)

Theme		Recommendation		n (%)
Professional biosafety				
	Use of PPE	Shoes for clinical use (surgical boots, closed work shoes with non-slip soles) [35,44,46,48,50,55]		6 (22.2)
		High-performance filtering masks/ respirators (N95, PFF2, PFF3, or similar) [30-41,43-45,48,49,51,54-56]		21 (77.7)
		Appropriate clothing (lab coat, apron, clinical uniform, scrubs, surgical pajamas, protective cloak) [30-44,46,48,50-56]		24 (88.8)
		Visor/face shield [30-41,43-45,47-50,52-54,56]		23 (85.2)
		Clinical PPE (cap, fluid-resistant face mask, gloves, goggles, shoe covers) [30-56]		27 (100.0)
	Antisepsis measures	Drying hands with disposable towels [36,50]		2 (7.4)
		Washing other exposed body parts (face, neck, ears) [30,34,38]		3 (11.1)
		Hand antisepsis with alcoholic gel (60 to 95%) [36-38,45,52,53,56]		7 (25.9)
		Hand washing (with water and soap) [30,34-38,40-42,44,45,48,50-52,56]		16 (59.2)
	Instructions on behavior and preventive measures (for managers and staff)	Avoiding handshakes [40]		1 (3.7)
		Wearing a conventional mask over the N95 respirator, allowing its reuse [32]		1 (3.7)
		Discarding PPE in the appropriate place [45]		1 (3.7)
		Not touching one’s mask [35]		1 (3.7)
		Replacing a long-sleeved apron with pajamas that cover the entire body [32]		1 (3.7)
		Daily washing of work clothes at a temperature of at least 60°C/140°F [35]		1 (3.7)
		Providing an appropriate place to put on the work uniform [49]		1 (3.7)
		Keeping nails natural, short and clean and not using artificial ones [50,55]		2 (7.4)
		Avoiding having a beard and /or mustache [44,4]		2 (7.4)
		Following cough/sneeze etiquette [30,42,51]		3 (11.1)
		Keeping updated inventories of available biosafety supplies [50,51]		2 (7.4)
		Using PPE to perform cleaning and disinfection [31,35,46]		3 (11.1)
		Monitoring professionals’ health to detect respiratory infection [36,38-40]		4 (14.8)
		Providing information and training on the adopted protocols [35,39,45,46]		4 (14.8)
		Providing the recommended immunization for healthcare professionals [39,40,47,51,53]		5 (18.5)
		Releasing professionals who belong to at-risk groups or have respiratory symptoms from the face-to-face work at the clinic [35,43,47,51,54]		5 (18.5)
		Not using or removing props [38,44,45,48,50,51,55]		7 (25.9)
Patients’/ companions’ safety				
	Supply of PPE for patients	Shoe covers [50,51]		2 (7.4)
		Disposable bib [31,39,48]		3 (11.1)
		Goggles [39,48,46,56]		4 (14.8)
		Masks [31-33,35,37,38,46,48,50,55,56]		11 (40.7)
	Supply of PPE for companions	Shoe covers [50]		1 (3.7)
		Goggles [41,48,56]		3 (11.1)
		Masks [31-33,38,41,48,50,54,56]		9 (33.3)
	Patients’ antisepsis measures	Pre-procedure mouthwash with 0.005 % to 0.1% cetylpyridinium chloride [39]		1 (3.7)
		Performing oral hygiene with proper brushing [39]		1 (3.7)
		Pre-procedure mouthwash with 0.2 % to 2% povidone-iodine [30,32,34,35,37-39,41,43,47-53]		15 (59,2)
		Hand washing/antisepsis (using water and soap, alcoholic gel 70 % to 80%) [31-35,38,39,41,42,44-46,50,52,53,56]		16 (59.2)
		Pre-procedure mouthwash with 0.5 % to 1.5% hydrogen peroxide [30-39,41-44,47,54]		22 (81.5)
	Screening on arrival/scheduling	Scheduling patients over 60 for the first appointment time in the morning [53]		1 (3.7)
		Keeping a record of all personal details reported by patients [50]		1 (3.7)
		Scheduling the patient or referring according to the classification of risk of COVID-19 infection [35]		1 (3.7)
		Making appointments only at scheduled times [35]		1 (3.7)
		Measuring the companion’s body temperature [36]		1 (3.7)
		Controlling appointment times while maintaining punctuality so as not to interfere with other patients' schedules [49]		1 (3.7)
		Scheduling aerosol-generating procedures for the last appointment of the day [49]		1 (3.7)
		Scheduling appointments with a time interval wide enough to minimize possible contact between patients [47,50]		2 (7.4)
		Measuring body temperature using a non-contact digital thermometer [41,45,46]		3 (11.1)
		Rescheduling or postponing care for patients with fever or responses suggestive of contamination [31,37,41,42,55]		5 (18,5)
		Performing screening to identify people potentially suspected of COVID-19 infection [30,37,41,42,49,56]		6 (22.2)
		Performing screening using technological tools (phone calls, video conferencing, text messaging, e-mails) to identify people potentially suspected of COVID-19 infection [31-35,43,47,48,50-52,54]		12 (44,4)
	Instructions on behavior and preventive measures	Encouraging patients to maintain respiratory etiquette [52]		1 (3.7)
		Isolating symptomatic patients [52]		1 (3.7)
		Providing masks for patients with instructions for use [38]		1 (3.7)
		Advising patients not to touch office surfaces [56]		1 (3.7)
		Asking patients to arrive on time [35,52]		2 (7.4)
		Recommending that patients avoid props [49,51]		2 (7.4)
		Preferably making payments without contact with cash (by credit card, cell phone) [35,46]		2 (7.4)
		Following cough/sneeze etiquette [37,42,52]		3 (11.1)
Organization and biosafety of the physical dental facility environment				
		Using plastic containers to store patients' belongings [49]		1 (3.7)
		Isolating patients with suspected or confirmed COVID-19 in private rooms with closed doors and private bathrooms [52]		1 (3.7)
		Providing a sink and soap for patients to clean their hands and face [37,38,42]		3 (11.1)
		Providing disposable wipes [30,37,54]		3 (11.1)
		Using a physical barrier (acrylic, glass or other) in any area where there is proximity between staff and patients [30,33,46,50]		4 (14.8)
		Avoiding companions [33,34,36,39,41-43,46-49,51]		12 (44.4)
		Avoiding patients staying in the waiting room [32,33,35,39,41,45-47,49,51,52,56]		12 (44.4)
		Removing objects that can enable spread of the virus (e.g., magazines, business cards, toys, etc.) [30-32,34,35,41-47,49-52]		16 (59,2)
		Keeping a safe distance (1-2 meters) [30,32-36,39-47,49-52,56]		20 (74.0)
	Washrooms	Making disposable hygiene items available (for example: soap and paper towels) [42]		1 (3.7)
		Not allowing tooth or removable prosthesis hygiene in the sink [49]		1 (3.7)
		Providing a pedal-operated trash bin for disposing of tissue paper [37,42,54]		3 (11.1)
	Dental care room	Removing curtains [49]		1 (3.7)
		When dealing with suspected cases of COVID-19, only the patient and the dentist should remain in the room [34]		1 (3.7)
		Avoiding nebulizers or aromatherapy instruments [41]		1 (3.7)
		Not using the cuspidor bowl [35,38]		2 (7.4)
		Using an air purifier (HEPA; high-efficiency particulate air [filter]) [46,49,50]		3 (11.1)
		Implementing physical barriers between dental units and/or maintaining distance of 1.5-2 meters between them [39,50,51]		3 (11.1)
		Preparing all necessary materials in advance and making available only those that will be used [34,35,55,56]		4 (14.8)
		Using waterproof disposable protectors on equipment and surfaces [35,45,48,50,51,56]		6 (22.2)
	Cleaning, disinfection, and antisepsis measures	Cleaning cell phones and bags or disinfecting shoes on a disinfectant mat at the entrance door [38]		1 (3.7)
		Providing the patient with a nebulizer with disinfectant to apply to the shoes [46]		1 (3.7)
		Cleaning and disinfecting reusable PPE (face shields and goggles) [39,47,56]		4 (14.8)
		Disinfecting received and/or sent packaging and materials [30,39,49,51,55]		5 (18.5)
		Autoclaving all critical instruments, including high-speed turbines and low-speed handpieces [30,36,37,39,42,47,48,50,56]		9 (33.3)
		Recommended surface cleaners/disinfectants/bactericides:		
			Benzalkonium chloride (0.1%) [39]	1 (3.7)
			Glutaraldehyde (2%) [30,49]	2 (7.4)
			Chlorine-based disinfectant [32,35,50]	3 (11.1)
			Quaternary ammonium-based disinfectant [30,38,48,49]	4 (14.8)
			Hydrogen peroxide (0.5%) [34,36,41,49,52]	5 (18.5)
			Other disinfectants [35,39,48,55,56]	5 (18.5)
			Soap/detergent [36,39,41,47,48,52,53,55,56]	9 (33.3)
			Alcohol (60 to 80%) [30,34-38,41,43,48,50,52,53,55,56]	14 (51.8)
		Sodium hypochlorite (0.1% or more) [32,34,36-39,41,43,45,48-50,52,53,55,56]		16 (59.2)
	Other preventive measures	Keeping contact files of all people entering the clinic, including email and phone number [50]		1 (3.7)
		Replacing the door handles with those that can be opened easily by pushing or pulling [30]		1 (3.7)
		Providing an exclusive area for the staff to change usual clothes and put on PPE [48]		1 (3.7)
		Not consuming food and drinks, putting on contact lenses, or applying makeup inside the dental clinic [51]		1 (3.7)
		Reserving an exclusive area for the removal of PPE and disinfection [39]		1 (3.7)
		Adding clearly visible marks on the floor to help patients maintain social distance [30,33]		2 (7.4)
		Providing hand-washing instructions [41,42,49,52]		4 (14.8)
		Avoiding crowds [34,36,41,42,44,55]		6 (22.2)
		Providing instructions on the prevention of contagion from COVID-19 and cough/sneeze etiquette [30,33,37-39,41,42,47,50-53]		12 (44.4)
		Providing alcohol for hand disinfection [30,35,37,39,41,42,47,49,52,54,55]		11 (40.7)
		Ensuring frequent renewal of natural air and avoiding air conditioning [30,31,34,35,39,41,46,48-51,55,56]		13 (48.1)
Work process in dental care				
		Using teledentistry to manage care/refer patients based on clinical criteria [40]		1 (3.7)
		Performing postoperative support after urgent or emergency care by phone, to avoid contact with the patient [36]		1 (3.7)
		Conducting consultations by call center whenever possible [52]		1 (3.7)
		Performing electronic prescriptions for high-risk patients [52]		1 (3.7)
		Transitioning to minimally invasive treatments [32,40,41,45]		4 (14.8)
		Avoiding the use of rotary instruments (high-speed turbines and low-speed handpieces) [32,33,37,41,43]		5 (18.5)
		Using a turbine equipped with a non-return system to limit the risk of cross-contamination [36,42,43,47,53-55]		7 (25.9)
		Using manual or low-speed rotating devices [36-38,47-49,51,52]		8 (29.6)
		Performing sutures with absorbable material [36-38,47,49,53-55]		8 (29.6)
		Performing extraoral radiographs preferentially to periapical radiographs [35,37,41,42,44,48,49,51,53,55]		10 (37.0)
		Avoiding the use of devices that generate aerosols such as bicarbonate jets and ultrasound [31,37,40,41,49,51-55]		10 (37.0)
		Postponing elective care and prioritizing urgent or emergent cases [32,33,35,36,39,44,48,50,52-54]		11 (40.7)
		Avoiding the use of dental syringes, especially in its mist form (spray) [31,35,36,37,41,47-49,51-54]		12 (44.4)
		Performing 4-handed procedures [30,34,36,37,40,43,44,46-51,53]		14 (51.8)
		Using absolute isolation with a rubber dam [30,31,34,36,37,41-44,46-52,55,56]		18 (66.6)
		Using high-power suction or aspiration to reduce the amount of saliva in the oral cavity [30-37,40,41,44,46-56]		22 (81.4)

COVID-19, coronavirus disease 2019; PPE, personal protective equipment.

**Table 3.** Standardized scores of each domain by the AGREE II assessment for the COVID-19 biosafety guidelines for dental practice included in the systematic review (n=27)

Author organization (country)	Domain (% adequacy)^1^						
	1. Scope and purpose	2. Stakeholder involvement	3. Rigor of development	4. Clarity of presentation	5. Applicability	6. Editorial independence	Quality of the guideline
The South African Dental Association (South Africa)	100	66.7	50.0	50.0	41.7	20.8	5
Institute of German Dentists (Germany)	100	50.0	41.7	83.3	35.4	0.0	6
Latin American Association of Pediatric Dentistry (Latin America)	72.2	22.2	6.2	50.0	2.0	4.2	4
Austrian Dental Association (Austria)	61.1	44.4	16.7	69.4	25.0	25.0	4
Ministry of Health (Argentina)	38.9	66.7	9.3	55.5	10.4	0.0	4
Dentistry Council (Belgium)	72.2	58.3	39.6	72.2	50.0	16.7	5
Federal Council of Dentistry (Brazil)	100	52.8	27.1	83.3	43.7	25.0	6
Ministry of Health, National Health Surveillance Agency (Brazil)	58.3	63.9	30.2	52.8	41.7	20.8	5
Straumann Group (Brazil)	75.0	55.5	29.1	69.4	31.2	20.8	5
Colombian Association of Faculties of Dentistry, Colombian Federation of Dentistry and Colombian Society of Dental Surgeons (Colombia)	97.2	58.3	21.9	61.1	31.2	20.8	5
Ministry of Health and Social Protection (Colombia)	94.4	55.5	25.0	63.9	39.6	25.0	4
College of Dental Surgeons (Costa Rica)	75.0	80.5	4.2	75.0	25.0	0.0	4
Ministry of Public Health (Costa Rica)	72.2	27.8	2.0	66.7	0.0	0.0	4
School of Dentistry, University of Chile (Chile)	83.3	63.9	6.2	66.7	0.0	0.0	4
Ministry of Health (Chile)	69.4	30.5	3.1	50.0	20.8	0.0	3
Collegiate Organization of Dentists of Spain (Spain)	91.6	30.5	29.1	83.3	45.8	0.0	5
Official College of Dentists and Stomatologists of Catalonia (Spain)	100	66.7	51.0	97.2	39.6	20.8	6
American Dental Association (USA)	44.4	25.0	4.2	88.9	0.0	0.0	4
Ministry of Public Health (Ecuador)	69.4	44.4	9.3	66.7	0.0	0.0	5
Stomatological School of Guatemala (Guatemala)	94.4	33.3	12.5	61.1	0.0	0.0	4
Indian Dental Association (India)	63.9	30.5	7.3	86.1	12.5	0.0	4
School of Dentistry, National Autonomous University of Mexico (Mexico)	77.7	38.9	5.2	61.1	0.0	0.0	4
Ministry of Health (New Zealand)	77.8	19.4	5.2	44.4	16.7	0.0	4
Ministry of Public Health and Social Welfare 05.07.2020 (Paraguay)	91.6	36.1	16.7	66.7	10.4	0.0	5
Ministry of Public Health and Social Welfare 04.14.2020 (Paraguay)	72.2	27.8	0.0	66.7	16.7	0.0	4
The Directorate-General of Health (Portugal)	55.5	52.8	14.6	63.9	52.8	20.8	4
Dental Association of Thailand (Thailand)	55.5	22.2	10.1	66.7	0.0	0.0	4

AGREE, Appraisal of Guidelines for Research and Evaluation; COVID-19, coronavirus disease 2019.

1 Percentage of maximum possible score per domain.

## SUPPLEMENTARY MATERIALS

Supplementary Material 1. List of researched associations and institutions (gray literature)

Supplementary Material 2. Search strategies used in databases

Supplementary Material 3. List of studies excluded and reasons for exclusion.

Supplementary Material 4. Detailed mean scores per item and adequacy assessment using Appraisal of Guidelines for Research and Evaluation (AGREE) II for the COVID-19 biosafety guidelines for dental practice included in the systematic review (n = 27).
