# Supplementary material for: Quality of biosafety guidelines for dental clinical practice throughout the world in the early COVID-19 pandemic: a systematic review
Source: Epidemiol Health. 2021 Oct 22;43:e2021089. doi: 10.4178/epih.e2021089 (PMC8920742; doi:10.4178/epih.e2021089)
Supplement: Supplementary Material 1. — List of researched associations and institutions (gray literature) [file epih-43-e2021089-suppl1.docx]

**Supplementary Material 1. List of researched associations and institutions (gray literature)**

| **Institution** | **Link** |
| --- | --- |
| Armenian Dental Association | <http://www.adaorg.am/> |
| Asociación Odontológica Uruguaya | <http://www.aou.org.uy/> |
| Dental Association of Bosnia & Herzegovina | <http://stomatoloskakomora.ba/> |
| Iranian Dental Association | <http://www.idaweb.ir/web/index.php> |
| Latvian Dental Association | <http://www.lza-zobi.lv/> |
| AFP Dental Society | <http://www.mydentista.com/AFPDS/AFPDentalSociety.htm> |
| American Association of Endodontists | <http://www.aae.org/> |
| American Association of Oral and Maxillofacial Surgeons | <http://www.aaoms.org/> |
| American Association of Orthodontists | <http://www.braces.org/> |
| American Dental Association | <https://www.ada.org/em> |
| American Veterinary Dental College | <http://www.avdc.org/> |
| Asociación Dental Mexicana | [http://www.adm.org.mx](http://www.adm.org.mx/) |
| Asociación Odontológica Dominicana | <https://aod.org.do/> |
| Asociación Odontológica Panameña | <http://www.aopan.org/> |
| Associaçao Brasileira de Odontología | <http://www.abo.org.br/> |
| Associação Brasileira de Radiologia Odontológica | [http://www.abro.org.br](http://www.abro.org.br/) |
| Associatie Nederlandse Tandartsen | [http://www.ant-online.nl](http://www.ant-online.nl/) |
| Association Dentaire Française | <http://www.adf.asso.fr/> |
| Association des Chirurgiens-Dentistes du Bénin | <https://www.lecourrierdudentiste.com/societes-scientifiques/association-des-chirurgiens-dentistes-du-benin-acdb.html> |
| Association for Dental Sciences of the Republic of China | <http://www.ads.org.tw/> |
| Association Marocaine de Prévention Bucco-Dentaire | <http://www.ampbd.com/> |
| Association of Dentists in Bulgaria | <http://bzs.bg/> |
| Association of Philippine Orthodontists | [http://apo.com.ph](http://apo.com.ph/) |
| Association of Prosthodontists of Canada | <http://www.prosthodontics.ca/> |
| Association of Public Health Dentists in Denmark | <http://www.tnl.dk/> |
| Associazione Italiana Odontoiatri | <http://www.aio.it/> |
| Associazione Nazionale Dentisti Italiani | <http://www.andi.it/> |
| Ateneo Argentino de Odontología | <http://www.ateneo-odontologia.org.ar/> |
| Australian Dental Association Inc | <http://www.ada.org.au/> |
| Austrian Dental Chamber | <http://www.zahnaerztekammer.at/> |
| Azeri Orthodontists Society | <http://www.orthodontics.az/> |
| Bahrain Dental Society | <http://www.bahraindental.com/> |
| Bangladesh Dental Society | [https://bangladeshdentalsociety.com](https://bangladeshdentalsociety.com/) |
| Botswana Dental Association | <https://www.bodeabw.com/> |
| British Dental Association | <http://www.bda.org/> |
| British Orthodontic Society | [http://www.bos.org.uk](http://www.bos.org.uk/) |
| British Society of Paediatric Dentistry | <http://www.bspd.co.uk/> |
| Bundeszahnärztekammer | <http://www.bzaek.de/> |
| Cambodian Dental Association | <http://www.dentistrycambodia.com/> |
| Canadian Academy of Pediatric Dentistry | <http://www.capd-acdp.org/> |
| Canadian Academy of Periodontology | <http://www.cap-acp.ca/> |
| Canadian Association of Oral and Maxillofacial Surgeons | <http://www.caoms.com/> |
| Canadian Association of Public Health Dentistry | <http://www.caphd-acsdp.org/> |
| Canadian Dental Association | <http://www.cda-adc.ca/> |
| Chambres Syndicales Dentaires | <http://www.incisif.org/> |
| Chinese Stomatological Association | [http://www.cndent.com](http://www.cndent.com/) |
| Chinese Taipei Association for Dental Sciences | <http://www.ads.org.tw/> |
| Colégio Brasileiro de Cirurgia e Traumatologia Bucomaxilofacial | [http://www.bucomaxilo.org.br](http://www.bucomaxilo.org.br/) |
| Colegio de Cirujano Dentistas de Chile | <http://www.colegiodentistas.cl/inicio/> |
| Colegio de Cirujanos Dentistas de Costa Rica | <http://colegiodentistas.org/> |
| Colegio de Odontólogos de Bolivia | <http://colodontologoslapaz.com/sociedades.php> |
| Colegio de Odontólogos de Venezuela | <http://www.elcov.org/> |
| **Appendix 1 continued**  Colegio Odontológico del Perú | <http://www.cop.org.pe/> |
| Collegi d-Odontolegs I Estomatolegs | <http://www.coec.cat/es/> |
| Confederación Odontológica de la República Argentina | <http://www.cora.org.ar/> |
| Consejo General de Colegio de Odontólogos y  Estomatólogos de España | <http://www.consejodentistas.org/> |
| Conselho Federal de Odontologia | <https://website.cfo.org.br/> |
| Croatian Dental Chamber | <http://www.hsk.hr/> |
| Croatian Dental Society | <http://www.hsd-cds.com/> |
| Cyprus Dental Association | <http://www.dental.org.cy/> |
| Czech Dental Chamber | <http://www.dent.cz/> |
| Dental Association of Thailand | <https://www.thaidental.or.th/> |
| Dental Chamber of Macedonia | <http://www.stomatologija.org.mk/skm/default.asp?nka=zaskm&jazik=em> |
| Dental Council of India | <http://www.dciindia.org/> |
| Dental Education India | <http://www.educationindiainfo.com/> |
| Dental Health Foundation | <http://www.dentalhealth.ie/> |
| Dutch association of dental hygienists | [http://www.mondhygienisten.nl](http://www.mondhygienisten.nl/) |
| Dutch association of denturists | [http://www.ont.nl](http://www.ont.nl/) |
| Dutch association of wholesalers in dentistry | [http://www.vgt.nl](http://www.vgt.nl/) |
| Egyptian Dental Association | <http://www.eda-egypt.org/> |
| Endodontic Society of the Philippines | <https://www.endodonticsociety.ph/> |
| Estonian Dental Association | <http://www.eststom.org.ee/> |
| European Veterinary Dental College | <http://www.evdc.info/> |
| FDI World Dental Federation | <http://www.fdiworldental.org/> |
| Federación Odontológica Colombiana | <http://www.encolombia.com/medicina_odont.htm> |
| Fiji Dental Association | <https://fijida.org/> |
| Finnish Dental Association | <http://www.hammaslaakariliitto.fi/> |
| Georgian Stomatological Association | <http://www.gsa.org.ge/> |
| German Dental Association | [http://www.deutsche-zahnarztauskunft.de](http://www.deutsche-zahnarztauskunft.de/) |
| Ghana Dental Association | <https://www.gdaonline.org/> |
| Hellenic Dental Association | <http://www.eoo.gr/> |
| Hungarian Dental Association | <http://www.mfe-hda.hu/> |
| Icelandic Dental Association | <http://www.tannsi.is/> |
| Indian Dental Association | <http://www.ida.org.in/> |
| Indian Orthodontic Society | <http://www.indianorthodonticsociety.org/> |
| Indonesian Dental Association | <http://www.pdgi-online.com/> |
| Iraqi Dental Association | <http://iraqidental.org/englishpage.htm> |
| Irish Dental Association | <http://www.dentist.ie/> |
| Israel Dental Association | [http://www.ida.org.il](http://www.ida.org.il/) |
| Japan Dental Association | <http://www.jda.or.jp/> |
| Jordan Dental Association | <http://www.jda.org.jo/> |
| Kenya Dental Association | <https://www.kda.or.ke/> |
| Korean Dental Association | <http://www.kda.or.kr/> |
| Lebanese Dental Association | <http://www.lda.org.lb/> |
| Lithuanian Dental Chamber | <http://www.odontologija.lt/> |
| Macedonian Dental Association | [http://msd.org.mk](http://msd.org.mk/) |
| Makati Dental Society | <http://filipinodentist.com/makatidentalsociety.htm> |
| Malaysian Dental Association | <http://www.mda.org.my/> |
| Malta Association of Dental Students | [http://www.madsonline.org](http://www.madsonline.org/) |
| Mongolian Dental Association | <http://shud.ucoz.com/> |
| Myanmar Dental Association | <http://www.mda-myanmar.org/> |
| National Union of Dentistry Societies | [http://www.unas.ro](http://www.unas.ro/) |
| Nederlandse Maatschappij tot Bevordering der Tandheelkunde | [http://www.nmt.nl]](about:blank) |
| Nepal Dental Association | <http://www.nda.org.np/> |
| New Zealand Dental Association | <http://www.nzda.org.nz/> |
| **Appendix 1 continued**  Nigerian Dental Association | <https://nigdentalasso.org/> |
| Norwegian Dental Association | <http://www.tannlegeforeningen.no/> |
| Ordem dos Médicos Dentistas | <http://www.omd.pt/> |
| Pakistan Dental Association | <http://www.pda.org.pk/> |
| Philippine Academy of General Dentistry | <http://www.mydentista.com/PAGD/PhilippineAcademyofGeneralDentistry.htm> |
| Philippine Academy of Implant Dentistry | <http://filipinodentist.com/paid.htm> |
| Philippine Dental Association | [http://www.pda.ph](http://www.pda.ph/) |
| Philippine Prosthodontic Society | <http://filipinodentist.com/prosthodonticsociety.htm> |
| Polish Chamber of Physicians and Dentists | <http://www.nil.org.pl/> |
| Polish Dental Society | <http://www.pts.net.pl/> |
| Romanian Dental Association of Private Practitioners | <http://www.dental.ro/> |
| Russian Dental Association | <http://www.e-stomatology.ru/> |
| Singapore Dental Association | <http://www.sda.org.sg/> |
| Sociedade Brasileira de Estomatologia e Patologia Oral | [http://www.estomatologia.com.br](http://www.estomatologia.com.br/) |
| Sociedade Brasileira de Pesquisa Odontológica | [http://www.sbpqo.org.br](http://www.sbpqo.org.br/) |
| Sociedade Portuguesa de Estomatologia e Medicina Dentária | <http://www.spemd.pt/> |
| Société de Médecine Dentaire | <http://www.dentiste.be/> |
| Société Suisse d'Odonto-Stomatologie | [http://www.sso.ch](http://www.sso.ch/) |
| South African Dental Association | <http://www.sadanet.co.za/> |
| Sri Lanka Dental Association | <http://www.slda.net/> |
| Swedish Dental Association | <https://tandlakarforbundet.se/in-english/> |
| Taiwan Academy of Pediatric Dentistry | <http://www.tapd.org.tw/> |
| Taiwan Association of Orthodontists | [http://www.tao.org.tw](http://www.tao.org.tw/) |
| Taiwan Dental Association | <http://www.cda.org.tw/> |
| The Belarussian Stomatological Association | <http://www.suvison.com/net/sc_aww_fp.asp?id=7796> |
| The Danish Dental Association | <http://www.dtfnet.dk/> |
| The Dental Council of Hong Kong | <http://www.dchk.org.hk/> |
| The Hong Kong Dental Association | [http://www.hkda.org](http://www.hkda.org/) |
| The Namibian Dental Association | <https://namibiadent.com/> |
| The Public Academy of Dentistry of the Republic of Moldova | <http://www.dentist.md/> |
| The Saudi Dental Society | <http://sdssa.org/> |
| The Stomatological Society of Greece | <http://www.stomatologia.gr/> |
| Turkish Dental Association | <http://www.tdb.org.tr/> |
| Uganda Dental Association | <https://www.ugadent.org/> |
| United Arab Emirates Medical Association | <http://www.aeedc.com/> |
| Universidad de Costa Rica: Facultad Odontologica | <http://www.ucr.ac.cr/clinica_odontologia.php> |
| Universidad Latina de Costa Rica | <http://www.ulatina.ac.cr/> |
| Verbond der Vlaamse Tandartsen | <http://www.tandarts.be/> |
| Zimbabwe Dental Association | <http://www.zida.org.zw/> |
