# Supplementary material for: Quality of biosafety guidelines for dental clinical practice throughout the world in the early COVID-19 pandemic: a systematic review
Source: Epidemiol Health. 2021 Oct 22;43:e2021089. doi: 10.4178/epih.e2021089 (PMC8920742; doi:10.4178/epih.e2021089)
Supplement: Supplementary Material 2. — Search strategies used in databases [file epih-43-e2021089-suppl2.docx]

**Supplementary Material 2. Search strategies used in databases**

| **PUBMED – may 06, 2020** | **128 Results** |
| --- | --- |
| # 1 Search ((((((((("coronavirus"[MeSH Terms] OR coronavirus[Title/Abstract]) OR Coronaviruses[Title/Abstract]) OR "severe acute respiratory syndrome coronavirus 2"[Title/Abstract]) OR "2019-nCoV"[Title/Abstract]) OR "Wuhan coronavirus"[Title/Abstract]) OR "SARS-CoV-2"[Title/Abstract]) OR "2019 novel coronavirus"[Title/Abstract]) OR "COVID-19 virus"[Title/Abstract])) | #2 ((((((((((((((((((((((((((((((((((((((("dentists"[MeSH Terms] OR Dentists[Title/Abstract]) OR "dentistry"[MeSH Terms]) OR Dentistry[Title/Abstract]) OR "dental care"[MeSH Terms]) OR Dental Care[Title/Abstract]) OR "containment of biohazards"[MeSH Terms]) OR "Containment of Biohazards"[Title/Abstract]) OR "endodontists"[MeSH Terms]) OR Endodontists[Title/Abstract]) OR "oral and maxillofacial surgeons"[MeSH Terms]) OR "orthodontists"[MeSH Terms]) OR Orthodontists[Title/Abstract]) OR Dentist[Title/Abstract]) OR Prosthodontists[Title/Abstract]) OR Prosthodontist[Title/Abstract]) OR "Prosthetic Dentist"[Title/Abstract]) OR "Prosthetic Dentists"[Title/Abstract]) OR "Restorative Dentist"[Title/Abstract]) OR "Restorative Dentists"[Title/Abstract]) OR (("dentists"[MeSH Terms] OR "dentists"[All Fields] OR "dentist"[All Fields]) AND Pediatric[Title/Abstract])) OR "Pediatric Dentist"[Title/Abstract]) OR "Pediatric Dentists"[Title/Abstract]) OR Periodontists[Title/Abstract]) OR Periodontist[Title/Abstract]) OR "pediatric dentistry"[MeSH Terms]) OR "Pediatric Dentistry"[Title/Abstract]) OR "public health dentistry"[MeSH Terms]) OR "Public Health Dentistry"[Title/Abstract]) OR "endodontics"[MeSH Terms]) OR Endodontics[Title/Abstract]) OR "orthodontics"[MeSH Terms]) OR Orthodontics[Title/Abstract]) OR "periodontics"[MeSH Terms]) OR Periodontics[Title/Abstract]) OR "prosthodontics"[MeSH Terms]) OR Prosthodontics[Title/Abstract]) OR "surgery, oral"[MeSH Terms]) OR "Surgery, Oral"[Title/Abstract]) OR "Care, Dental"[Title/Abstract]) OR Biosafety[Title/Abstract]) |
| #1AND#2 | |
| **LILACS E BBO (via BVS)**  **- May 05, 2020** | **195 Results** |
| #1 tw:((tw:("Infecções por Coronavirus")) OR (tw:("Infecciones por Coronavirus")) OR (tw:("Coronavirus Infections")) | #2  (tw:("Dentists")) OR (tw:("Odontólogos")) OR (tw:("Endodontia")) OR (tw:("Endodoncia")) OR (tw:("Endodontics")) OR (tw:("Orthodontics")) OR (tw:("Ortodoncia")) OR (tw:("Ortodontia")) OR (tw:("Periodontics")) OR (tw:("Periodoncia")) OR (tw:("Periodontia")) OR (tw:("Geriatric Dentistry")) OR (tw:("Odontología Geriátrica")) OR (tw:("Odontologia Geriátrica")) OR (tw:("Oral Surgical Procedures")) OR (tw:("Procedimientos Quirúrgicos Orales")) OR (tw:("Procedimentos Cirúrgicos Bucais")) OR (tw:("Odontopediatria")) OR (tw:("Odontología Pediátrica")) OR (tw:("Pediatric Dentistry")) OR (tw:("Dentistry")) OR (tw:("Odontología")) OR (tw:("Odontologia")) OR (tw:("Contenção de Riscos Biológicos")) OR (tw:("Containment of Biohazards")) OR (tw:("Contención de Riesgos Biológicos"))) AND ( db:("LILACS")) AND (year_cluster:[2019 TO 2020]) |
| #1AND#2 | |
| **WEB OF SCIENCE – May 06, 2020** | **118 Results** |
| # 1 TÓPICO: (dentist*) OR TÓPICO: ("Prosthetic dentist*") OR TÓPICO: ("Restorative dentist*") OR TÓPICO: ("Pediatric dentist*") OR TÓPICO: (Periodontist*) OR TÓPICO: ("Oral Medicine") OR TÓPICO: ("Preventive Dentistry") OR TÓPICO: (Endodontic*) OR TÓPICO: ("Dental Care") OR TÓPICO: ("Dental Health Services") OR TÓPICO: ("Containment of Biohazards") OR TÓPICO: (Biosafety) OR TÓPICO: ("Oral Maxillofacial Surgeons") OR TÓPICO: (Surgery) OR TÓPICO: (Orthodontic*) OR TÓPICO: ("Public Health Dentistry") | TÓPICO: (Coronavirus*) OR TÓPICO: ("severe acute respiratory syndrome coronavirus 2") OR TÓPICO: (SARS*) OR TÓPICO: (COVID*) OR TÓPICO: ("2019 nCoV") OR TÓPICO: ("Wuhan coronavirus") OR TÓPICO: ("2019 novel coronavirus") OR TÓPICO: ("SARS CoV 2") OR TÓPICO: ("COVID-19 virus") OR TÓPICO: ("coronavirus disease 2019") OR TÓPICO: ("Wuhan seafood market pneumonia virus") |
| #1AND#2 | |
| **SCOPUS – May 06, 2020** | **75 Results** |
| # 1 ( ( TITLE-ABS-KEY ( dentist ) OR TITLE-ABS-KEY ( "Prosthetic dentist" ) OR TITLE-ABS-KEY ( "Restorative dentist" ) OR TITLE-ABS-KEY ( "Pediatric dentist" ) OR TITLE-ABS-KEY ( periodontist ) OR TITLE-ABS-KEY ( dentist* ) OR TITLE-ABS-KEY ( "Oral Medicine" ) OR TITLE-ABS-KEY ( "Preventive Dentistry" ) OR TITLE-ABS-KEY ( endodontics ) OR TITLE-ABS-KEY ( "Dental Care" ) OR TITLE-ABS-KEY ( "Dental Health Services" ) OR TITLE-ABS-KEY ( "Containment of Biohazards" ) OR TITLE-ABS-KEY ( biosafety ) OR TITLE-ABS-KEY ( "Oral and Maxillofacial Surgeons" ) OR TITLE-ABS-KEY ( orthodontic ) OR TITLE-ABS-KEY ( "Public Health Dentistry" ) OR TITLE-ABS-KEY ( dent* ) ) | #2  ( ( TITLE-ABS-KEY ( coronavirus ) OR TITLE-ABS-KEY ( coronaviruses ) OR TITLE-ABS-KEY ( "severe acute respiratory syndrome coronavirus 2" ) OR TITLE-ABS-KEY ( "2019-nCoV" ) OR TITLE-ABS-KEY ( "Wuhan coronavirus" ) OR TITLE-ABS-KEY ( "SARS-CoV-2" ) OR TITLE-ABS-KEY ( covid* ) OR TITLE-ABS-KEY ( "2019 novel coronavirus" ) OR TITLE-ABS-KEY ( "COVID-19 virus" ) OR TITLE-ABS-KEY ( "coronavirus disease 2019 virus" ) OR TITLE-ABS-KEY ( "Wuhan seafood market pneumonia virus" ) ) ) AND ( LIMIT-TO ( PUBYEAR , 2020 ) OR LIMIT-TO ( PUBYEAR , 2019 ) ) |
| #1AND#2 | |
| **COCHRANE – May 06, 2020** | **3 Results** |
| #1 (Dentists):ti,ab,kw OR (Dentistry):ti,ab,kw OR (Dental Care):ti,ab,kw OR (Containment of Biohazards):ti,ab,kw OR (Endodontists):ti,ab,kw OR (Oral and Maxillofacial Surgeons):ti,ab,kw OR (Orthodontists):ti,ab,kw OR (Preventive Dentistry):ti,ab,kw OR (Endodontics):ti,ab,kw OR (Orthodontics):ti,ab,kw OR (Pediatric Dentistry):ti,ab,kw OR (Periodontics):ti,ab,kw OR (Prosthodontics):ti,ab,kw OR (Public Health Dentistry):ti,ab,kw OR (Surgery Oral):ti,ab,kw OR (Biohazards):ti,ab,kw OR (Prosthodontist*):ti,ab,kw OR (Prosthodontics):ti,ab,kw OR ("Dentist* Prosthetic"):ti,ab,kw OR ("Prosthetic* Dentist"):ti,ab,kw OR ("Dentist* Restorative"):ti,ab,kw OR ("Restorative Dentist*"):ti,ab,kw OR ("Dentist* Pediatric"):ti,ab,kw OR ("Pediatric Dentist*"):ti,ab,kw OR (Periodontist*):ti,ab,kw OR (Biosafety):ti,ab,kw OR ("Biohazard Containment"):ti,ab,kw | #2 (Coronavirus):ti,ab,kw OR (severe acute respiratory syndrome coronavirus 2):ti,ab,kw OR (Coronaviruses):ti,ab,kw OR ("2019-nCoV"):ti,ab,kw OR ("Wuhan coronavirus"):ti,ab,kw OR ("SARS-CoV-2"):ti,ab,kw OR ("2019 novel coronavirus"):ti,ab,kw OR ("COVID-19 virus"):ti,ab,kw OR ("coronavirus disease 2019 virus"):ti,ab,kw OR ("Wuhan seafood market pneumonia virus"):ti,ab,kw |
| #1AND#2 | |
