# Supplementary material for: Quality of biosafety guidelines for dental clinical practice throughout the world in the early COVID-19 pandemic: a systematic review
Source: Epidemiol Health. 2021 Oct 22;43:e2021089. doi: 10.4178/epih.e2021089 (PMC8920742; doi:10.4178/epih.e2021089)
Supplement: Supplementary Material 3. — List of studies excluded and reasons for exclusion. [file epih-43-e2021089-suppl3.docx]

**Supplementary Material 3. List of studies excluded and reasons for exclusion.**

| **Study** | **Reason for exclusion** |
| --- | --- |
| B. Ather, N.B Patel, A. Ruparel, A. Diogenes, K.M. Hargreaves, Coronavirus Disease 19 (COVID-19): Implications for Clinical Dental Care, J Endod. 46 (2020) 584-595. doi:10.1016/j.joen.2020.03.008 | Review/opinion article |
| S. Alharbi, S. Alharbi, S. Alqaidi. Guidelines for dental care provision during the COVID-19 pandemic [published online ahead of print, 2020 Apr 7],  Saudi Dent J. 32 (2020) 181-186. doi:10.1016/j.sdentj.2020.04.001 | Review/opinion article |
| Brazil. Ministry of Health. New Coronavirus Treatment Protocol (2019-nCoV). 2020https://portalarquivos2.saude.gov.br/images/pdf/2020/fevereiro/05/Protocolo-de-manejo-clinico-para-o-novo-coronavirus-2019-ncov.pdf (accessed 30 September 2020). | Biosafety measures in general for health professionals |
| Brazil. Ministry of Health. Dental care flowchart. 2020.https://website.cfo.org.br/ministerio-da-saude-atende-pedido-do-cfo-e-regulamenta-atendimento-odontologico-no-sus/ (accessed 30 September 2020). | Isolated flowchart of a protocol |
| Brazil. Ministry of Health. Coronavirus Covid-19 Dental care at SUS. 2020. <https://www.abo.org.br/uploads/files/2020/03/covid-19-atendimento-odontologico-no-sus.pdf> (accessed 30 September 2020). | Without references |
| C.S.[Verdugo,](https://pesquisa.bvsalud.org/portal/?lang=pt&q=au:%22Verdugo,%20Crist%C3%B3bal%20Sep%C3%BAlveda%22) A.S. [Álvarez,](https://pesquisa.bvsalud.org/portal/?lang=pt&q=au:%22%C3%81lvarez,%20Alfio%20Secchi%22) F.[Donoso-Hofer](https://pesquisa.bvsalud.org/portal/?lang=pt&q=au:%22Donoso-Hofer,%20Francisca%22), Considerations in the Dental Emergency Service in the Context of Coronavirus COVID-19 (SARS-CoV-2), [Int. j. odontostomatol. (Print)](http://portal.revistas.bvs.br/transf.php?xsl=xsl/titles.xsl&xml=http://catserver.bireme.br/cgi-bin/wxis1660.exe/?IsisScript=../cgi-bin/catrevistas/catrevistas.xis%7Cdatabase_name=TITLES%7Clist_type=title%7Ccat_name=ALL%7Cfrom=1%7Ccount=50&lang=pt&comefrom=home&home=false&task=show_magazines&request_made_adv_search=false&lang=pt&show_adv_search=false&help_file=/help_pt.htm&connector=ET&search_exp=Int.%20j.%20odontostomatol.%20(Print)). 14 (2020) 279-284. | Review/opinion article |
| R. L. Giudice. The Severe Acute Respiratory Syndrome Coronavirus-2 (SARS CoV-2) in Dentistry. Management of Biological Risk in Dental Practice. Int J Environ Res Public Health. 17 (2020);17(9):3067. Published 2020 Apr 28. doi:10.3390/ijerph17093067 | Review/opinion article |
| C.G. Hua, Z.Q Liu, Q. Wang, Z. Yang, Q.H. Xu, J. Zhang. Strategy of dental clinics to cope with the epidemic period of infectious diseases based on the experience of corona virus disease outbreak, [West China Journal of Stomatology](http://www.hxkqyxzz.net/). 38 (2020) 117-121. doi:10.7518/hxkq.2020.02.001 | Biosafety measures for the dental clinic and hospital environment. |
| French Society of Stomatology, Maxillo-Facial Surgery and Oral Surgery (SFSCMFCO), Practitioners specialized in oral health and coronavirus disease 2019: Professional guidelines from the French society of stomatology, maxillofacial surgery and oral surgery, to form a common front against the infectious risk, J Stomatol Oral Maxillofac Surg. 121 (2020) 155-158. doi:10.1016/j.jormas.2020.03.011. | Review/opinion article |
| H.S. Tang, Z.Q. Yao, W.M. Wang, Emergency management of prevention and control of novel coronavirus pneumonia in departments of stomatology Zhonghua Kou Qiang Yi Xue Za Zhi. 55 (2020) 246-248. doi:10.3760/cma.j.cn112144-20200205-00037 | Biosafety measures at the hospital level |
| A. Abramovitz, D. Palmon, Levy, et al. Dental care during the coronavirus disease 2019 (COVID-19) outbreak: operatory considerations and clinical aspects. Quintessence Int. 2020;51(5):418-429. doi:10.3290/j.qi.a44392 | Review/opinion article |
| P.R. Martins-Filho, V.T Gois-Santos, C.S.S. Tavares, E.G.M. Melo, E.M. Nascimento-Júnior, V.S. Santos, Recommendations for a safety dental care management during SARS-CoV-2 pandemic, Rev Panam Salud Publica. 44:e51 (2020). https://doi. org/10.26633/RPSP.2020.51 | Brief communication |
| Y.F. Ren, L. Rasubala, H. Malmstrom, E. Eliav. Dental Care and Oral Health under the Clouds of COVID-19, JDR Clin Trans Res. 5 (2020) 202-210. doi:10.1177/2380084420924385 | Special communication |
| Z. Zhao, D. Gao. Precaution of 2019 novel coronavirus infection in department of oral and maxillofacial surgery, Br J Oral Maxillofac Surg. (2020) 250-253. doi:10.1016/j.bjoms.2020.03.001 | Biosafety measures at the hospital level |
| X. Peng, X. Xu, Y. Li, L. Cheng, X. Zhou, B. Ren. Transmission routes of 2019-nCoV and controls in dental practice. International Journal of Oral Science. 12 (2020). https://doi.org/10.1038/s41368-020-0075-9 | Review/opinion article |
