# Supplementary material for: Quality of biosafety guidelines for dental clinical practice throughout the world in the early COVID-19 pandemic: a systematic review
Source: Epidemiol Health. 2021 Oct 22;43:e2021089. doi: 10.4178/epih.e2021089 (PMC8920742; doi:10.4178/epih.e2021089)
Supplement: Supplementary Material 4. — Detailed mean scores per item and adequacy assessment using Appraisal of Guidelines for Research and Evaluation (AGREE) II for the COVID-19 biosafety guidelines for dental practice included in the systematic review (n = 27). [file epih-43-e2021089-suppl4.docx]

**Supplementary Material 4.** Detailed mean scores per item and adequacy assessment using Appraisal of Guidelines for Research and Evaluation (AGREE) II for the

COVID-19 biosafety guidelines for dental practice included in the systematic review (n = 27).

|  | **Domain (% adequacy*/ average score per item)** | | | | | | | | | | | | | | | | | | | | | | | |  |
| --- | --- | --- | --- | --- | --- | --- | --- | --- | --- | --- | --- | --- | --- | --- | --- | --- | --- | --- | --- | --- | --- | --- | --- | --- | --- |
| **Author Organization (Country/Region)** | **1. Scope and Purpose** | | | **2. Stakeholder Involvement** | | | **3. Rigour of Development** | | | | | | | | **4. Clarity of Presentation** | | | **5. Applicability** | | | | | **6. Editorial Independence** | |  |
|  | **1** | **2** | **3** | **4** | **5** | **6** | **7** | **8** | **9** | **10** | **11** | **12** | **13** | **14** | **15** | **16** | **17** | **18** | **19** | **20** | **21** | | **22** | **23** |  |
| The South African Dental Association/ South Africa | **100.0** | | | **66.7** | | | **50.0** | | | | | | | | **50.0** | | | **41.7** | | | | | **20.8** | |  |
|  | 7 | 7 | 7 | 7 | 1 | 7 | 4 | 2 | 4 | 3 | 4 | 7 | 7 | 1 | 2 | 5 | 5 | 3 | 7 | 3 | 1 | | 1 | 1 |  |
| Institute of German Dentists/ Germany | **100.0** | | | **50.0** | | | **41.7** | | | | | | | | **83.3** | | | **35.4** | | | | | **0** | |  |
|  | 7 | 7 | 7 | 1 | 1 | 7 | 1 | 1 | 4 | 5 | 3 | 4 | 1 | 7 | 5 | 6 | 6 | 3 | 7 | 1 | 1 | | 1 | 1 |  |
| Latin American Association of Pediatric Dentistry/ Latin America | **72.2** | | | **22.2** | | | **6.2** | | | | | | | | **50.0** | | | **2.0** | | | | | **4.2** | |  |
|  | 6 | 4 | 3 | 1 | 1 | 5 | 1 | 1 | 1 | 1 | 1 | 3 | 1 | 1 | 4 | 3 | 4 | 1 | 1 | 1 | 1 | | 1 | 1 |  |
| Austrian Dental Association/ Austria | **61.1** | | | **44.4** | | | **16.7** | | | | | | | | **69.4** | | | **25.0** | | | | | **25.0** | |  |
|  | 1 | 6 | 7 | 1 | 1 | 7 | 4 | 2 | 1 | 1 | 1 | 4 | 1 | 1 | 7 | 4 | 6 | 1 | 4 | 1 | 1 | | 3 | 1 |  |
| Ministry of Health/ Argentina | **38.9** | | | **66.7** | | | **9.3** | | | | | | | | **55.5** | | | **10.4** | | | | | **0** | |  |
|  | 1 | 3 | 4 | 1 | 1 | 5 | 1 | 1 | 1 | 1 | 1 | 4 | 1 | 1 | 5 | 5 | 5 | 1 | 3 | 1 | 1 | | 1 | 1 |  |
| Dentistry Council/ Belgium | **72.2** | | | **58.3** | | | **39.6** | | | | | | | | **72.2** | | | **50.0** | | | | | **16.7** | |  |
|  | 1 | 7 | 7 | 4 | 1 | 7 | 2 | 1 | 1 | 3 | 7 | 7 | 1 | 1 | 7 | 7 | 4 | 4 | 7 | 1 | 4 | | 7 | 1 |  |
| Federal Council of Dentistry/ Brazil | **100.0** | | | **52.8** | | | **27.1** | | | | | | | | **83.3** | | | **43.7** | | | | | **25.0** | |  |
|  | 7 | 7 | 7 | 7 | 1 | 7 | 3 | 1 | 3 | 2 | 5 | 4 | 1 | 3 | 7 | 7 | 5 | 2 | 7 | 2 | 2 | | 1 | 1 |  |
| Ministry of Health/ National Health Surveillance Agency/ Brazil | **58.3** | | | **63.9** | | | **30.2** | | | | | | | | **52.8** | | | **41.7** | | | | | **20.8** | |  |
|  | 1 | 6 | 7 | 7 | 1 | 7 | 3 | 1 | 2 | 2 | 4 | 4 | 1 | 4 | 4 | 5 | 4 | 2 | 7 | 3 | 1 | | 6 | 1 |  |
| Straumann Group/ Brazil | **75.0** | | | **55.5** | | | **29.1** | | | | | | | | **69.4** | | | **31.2** | | | | | **20.8** | |  |
|  | 3 | 7 | 7 | 7 | 1 | 7 | 2 | 1 | 3 | 4 | 2 | 4 | 1 | 1 | 6 | 4 | 4 | 1 | 7 | 3 | 1 | | 4 | 1 |  |
| Colombian Association of Faculty of Dentistry, Colombian Federation of Dentistry y Colombian Society of Dental Surgeons/ Colombia | **97,2%** | | | **58,3%** | | | **21,8%** | | | | | | | | **61,1%** | | | **31,2%** | | | | | **20,8%** | |  |
|  | 7 | 7 | 7 | 7 | 1 | 6 | 2 | 2 | 3 | 2 | 4 | 4 | 1 | 1 | 6 | 5 | 4 | 2 | 7 | 2 | 1 | | 1 | 1 |  |
| Ministry of Health and Social Protection/ Colombia | **94,4%** | | | **55,5%** | | | **25%** | | | | | | | | **63,8%** | | | **39,5%** | | | | | **25%** | |  |
|  | 7 | 6 | 7 | 4 | 1 | 7 | 2 | 1 | 2 | 2 | 2 | 4 | 7 | 1 | 6 | 6 | 3 | 3 | 7 | 3 | 1 | | 1 | 1 |  |
| College of Dental Surgeons/ Costa Rica | **75.0** | | | **80.5** | | | **4.2** | | | | | | | | **75.0** | | | **25.0** | | | | | **0** | |  |
|  | 5 | 6 | 6 | 2 | 1 | 5 | 1 | 1 | 1 | 1 | 1 | 1 | 1 | 1 | 5 | 5 | 5 | 1 | 7 | 1 | 1 | | 1 | 1 |  |
| Ministry of Public Health/ Costa Rica | **72.2** | | | **27.8** | | | **2.0** | | | | | | | | **66.7** | | | **0** | | | | | **0** | |  |
|  | 5 | 5 | 6 | 1 | 1 | 6 | 1 | 1 | 1 | 1 | 1 | 1 | 1 | 1 | 5 | 4 | 5 | 1 | 1 | 1 | 1 | | 1 | 1 |  |
| School of Dentistry/University of Chile/ Chile | **83.3** | | | **63.9** | | | **6.2** | | | | | | | | **66.7** | | | **0** | | | | | **0** | |  |
|  | 5 | 5 | 6 | 7 | 1 | 6 | 1 | 1 | 1 | 1 | 1 | 4 | 1 | 1 | 5 | 5 | 5 | 1 | 1 | 1 | 1 | | 1 | 1 |  |
| Ministry of Health/ Chile | **69.4** | | | **30.5** | | | **3.1** | | | | | | | | **50.0** | | | **20.8** | | | | | **0** | |  |
|  | 6 | 4 | 5 | 1 | 1 | 6 | 1 | 1 | 1 | 1 | 1 | 3 | 1 | 1 | 5 | 3 | 4 | 1 | 6 | 1 | 1 | | 1 | 1 |  |
| Collegiate Organization of Dentists of Spain/ Spain | **91.6** | | | **30.5** | | | **29.1** | | | | | | | | **83.3** | | | **45.8** | | | | **0** | **91.6** | |  |
|  | 7 | 6 | 6 | 1 | 1 | 6 | 2 | 2 | 1 | 1 | 1 | 4 | 2 | 1 | 6 | 6 | 5 | 4 | 6 | 3 | 1 | | 1 | 1 |  |
| Official Collegi d'Odontòlegs i Estomatòlegs de Catalunya/ Spain | **100.0** | | | **66.7** | | | **51.0** | | | | | | | | **97.2** | | | **39.6** | | | | | **20.8** | |  |
|  | 7 | 7 | 7 | 7 | 1 | 7 | 1 | 6 | 4 | 4 | 7 | 7 | 4 | 1 | 7 | 7 | 7 | 3 | 7 | 1 | 3 | | 1 | 1 |  |
| American Dental Association/ United States | **44.4** | | | **25.0** | | | **4.2** | | | | | | | | **88.9** | | | **0** | | | | | **0** | |  |
|  | 1 | 3 | 5 | 1 | 1 | 5 | 1 | 1 | 1 | 1 | 1 | 3 | 1 | 1 | 7 | 6 | 5 | 1 | 1 | 1 | 1 | | 1 | 1 |  |
| Ministry of Public Health/ Ecuador | **69.4** | | | **44.4** | | | **9.3** | | | | | | | | **66.7** | | | **0** | | | | | **0** | |  |
|  | 5 | 5 | 5 | 1 | 1 | 5 | 1 | 1 | 1 | 1 | 1 | 3 | 1 | 1 | 5 | 3 | 5 | 1 | 1 | 1 | 1 | | 1 | 1 |  |
| Stomatological School of Guatemala/ Guatemala | **94.4** | | | **33.3** | | | **12.5** | | | | | | | | **61.1** | | | **0** | | | | | **0** | |  |
|  | 7 | 6 | 7 | 1 | 1 | 7 | 2 | 2 | 1 | 1 | 1 | 4 | 1 | 1 | 5 | 5 | 5 | 1 | 1 | 1 | 1 | | 1 | 1 |  |
| Indian Dental Association/ India | **63.9** | | | **30.5** | | | **7.3** | | | | | | | | **86.1** | | | **12.5** | | | | | **0** | |  |
|  | 1 | 6 | 7 | 1 | 1 | 6 | 1 | 1 | 1 | 1 | 1 | 5 | 1 | 1 | 6 | 6 | 6 | 1 | 5 | 1 | 1 | | 1 | 1 |  |
| School of Dentistry/ National Autonomous University of Mexico/ Mexico | **77.7** | | | **38.9** | | | **5.2** | | | | | | | | **61.1** | | | **0** | | | | | **0** | |  |
|  | 4 | 5 | 5 | 1 | 1 | 5 | 1 | 1 | 1 | 1 | 1 | 3 | 1 | 1 | 5 | 1 | 5 | 1 | 1 | 1 | 1 | | 1 | 1 |  |
| Ministry of Health/ New Zealand | **77.8** | | | **19.4** | | | **5.2** | | | | | | | | **44.4** | | | **16.7** | | | | | **0** | |  |
|  | 6 | 5 | 4 | 1 | 1 | 4 | 1 | 1 | 1 | 1 | 1 | 3 | 1 | 1 | 4 | 3 | 4 | 1 | 4 | 3 | 1 | | 1 | 1 |  |
| Ministry of Public Health and Social Welfare 07.05.2020/ Paraguay | **91.6** | | | **36.1** | | | **16.7** | | | | | | | | **66.7** | | | **10.4** | | | | | **0** | |  |
|  | 6 | 6 | 7 | 1 | 1 | 7 | 1 | 1 | 1 | 1 | 1 | 1 | 7 | 3 | 5 | 1 | 5 | 1 | 5 | 1 | 1 | | 1 | 1 |  |
| Ministry of Public Health and Social Welfare 14 .04.2020/ Paraguay | **72.2** | | | **27.8** | | | **0** | | | | | | | | **66.7** | | | **16.7** | | | | | **0** | |  |
|  | 4 | 5 | 5 | 1 | 1 | 5 | 1 | 1 | 1 | 1 | 1 | 1 | 1 | 1 | 5 | 4 | 5 | 1 | 5 | 1 | 1 | | 1 | 1 |  |
| The Directorate-General of Health/ Portugal | **55.5** | | | **52.8** | | | **14.6** | | | | | | | | **63.9** | | | **52.8** | | | | | **20.8** | |  |
|  | 1 | 5 | 5 | 4 | 1 | 5 | 1 | 1 | 2 | 1 | 5 | 2 | 1 | 1 | 6 | 6 | 3 | 1 | 6 | 1 | 1 | | 6 | 1 |  |
| Dental Association of Thailand/ Thailand | **55.5** | | | **22.2** | | | **10.1** | | | | | | | | **66.7** | | | **0** | | | | | **0** | |  |
|  | 2 | 5 | 5 | 1 | 1 | 5 | 1 | 1 | 1 | 1 | 1 | 4 | 1 | 1 | 5 | 5 | 4 | 1 | 1 | 1 | 1 | | 1 | 1 |  |

* Percentage of maximum possible score per domain.
